# *Grimontia indica* AK16^T^, sp. nov., Isolated from a Seawater Sample Reports the Presence of Pathogenic Genes Similar to *Vibrio* Genus

**DOI:** 10.1371/journal.pone.0085590

**Published:** 2014-01-21

**Authors:** Aditya Singh, Bhumika Vaidya, Indu Khatri, T. N. R. Srinivas, Srikrishna Subramanian, Suresh Korpole, Anil Kumar Pinnaka

**Affiliations:** 1 Microbial Type Culture Collection & Gene Bank, Institute of Microbial Technology, Chandigarh, India; 2 National Institute of Oceanography, Visakhapatnam, Andhra Pradesh, India; Institut National de la Recherche Agronomique, France

## Abstract

*Grimontia indica* strain AK16^T^ sp. nov. is the type strain of *G. indica* sp. nov. a new species within the genus *Grimontia*. This strain, whose genome is described here, was isolated from seawater sample collected from southeast coast of Palk Bay, India. *G. indica* AK16^T^ is a Gram-negative, facultative aerobic rod shaped bacterium. There are only two other strains in the genus *Grimontia* one of which, *Grimontia hollisae* CIP 101886^T^, is a reported human pathogen isolated from human stool sample while the other, ‘*Grimontia marina* IMCC5001^T^’, was isolated from a seawater sample. As compared to the pathogenic strain *Grimontia hollisae* CIP 101886^T^, the strain AK16^T^ lacks some genes for pathogenesis like the accessory colonization factors AcfA and AcfD, which are required for the colonization of the bacterium in the host body. While it carries some pathogenesis genes like OmpU, which are related to pathogenesis of *Vibrio* strains. This suggests that the life cycle of AK16^T^ may include some pathogenic interactions with marine animal(s), or it may be an opportunistic pathogen. Study of the *Grimontia* genus is important because of the severe pathogenic traits exhibited by a member of the genus with only three species reported in total. The study will provide some vital information which may be useful in future clinical studies on the genus.

## Introduction

**Figure 1:**
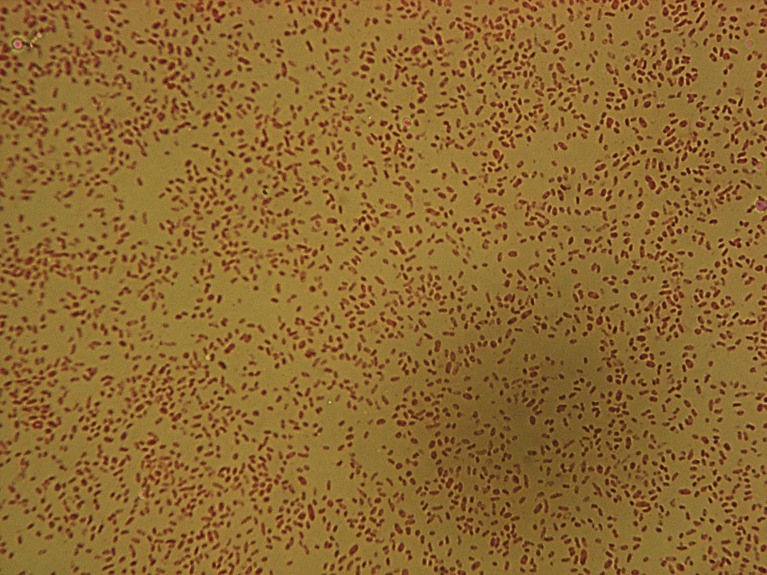
Gram staining of strain AK16^T^. Gram staining of strain AK16^T^ clearly represents a gram-staining-negative rod shaped bacterium.

**Figure 2:**
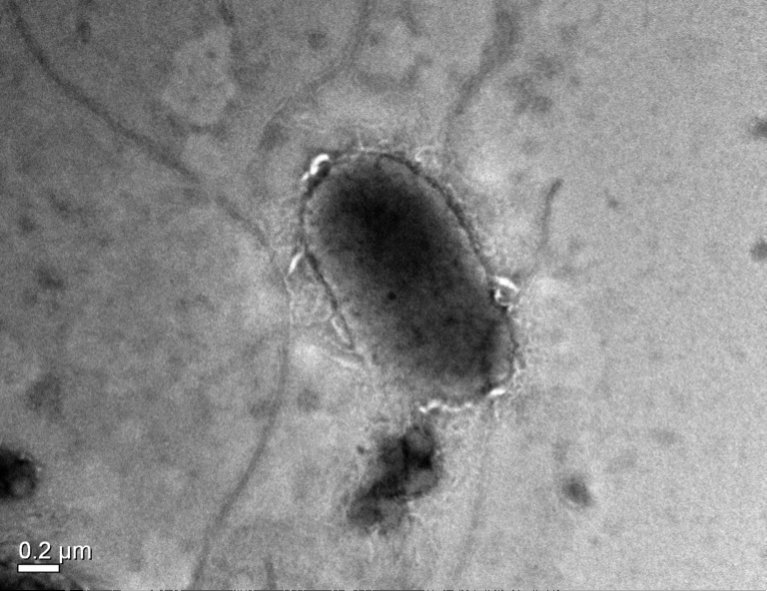
Transmission electron microscopy of the strain AK16^T^. Transmission electron microscopy of the strain AK16^T^ using Jeol JEM2100 at operating voltage of 200 kV. The bacterium is rod shaped with a monotrichous flagellum.

*Grimontia indica* strain AK16^T^ [ = JCM (Japan Collection of Microorganisms) 17852^T^ = MTCC (Microbial Type Culture Collection & Gene Bank) 11632^T^], is the type strain of *G. indica* sp. nov. This bacterium is a Gram-negative (Figure 1), facultative aerobic, motile with single monotrichous flagellum (Figure 2), rod-shaped, catalase and oxidase positive bacterium that was isolated from a seawater sample collected from southeast seacoast of Palk Bay, India. The major fatty acids were C_12:0_, C_12:0_ 3-OH, C_14:0_, C_14:0_ 3-OH and/or C_16:1_ iso-I, C_16:1_ *ω7c* and/or C_15:0_ iso 2-OH. For the current classification of the prokaryotes polyphasic taxonomy is used which depends on a combination of phenotypic and genotypic characteristics [1]. More than 3,000 bacterial genomes have been sequenced and annotated throughout the world till now, here we proposed to integrate genomic data in description of novel bacterial species [2]–[6].

The genus *Grimontia* was created in 2003 by reclassification of *Vibrio hollisae* as *Grimontia hollisae* [7]. It consists of Gram-negative, aerobic, motile, rod-shaped bacteria. The genus *Grimontia* belongs to the family *Vibrionaceae*, order *Vibrionales*. The genus currently consists of two reported species, *G. hollisae* CIP 101886^T^ being the first one to be found and also the type species, it was isolated from the stool sample of a diarrhea patient [7]. The other reported species, “*Grimontia marina* IMCC5001^T^”, was isolated from seawater sample collected from Yellow sea [8]. *G. hollisae* CIP 101886^T^, is reported to be pathogenic to humans, causing severe gastroenteritis and hypovolemic shock [9].

Whole genome sequencing of the strain AK16^T^ was performed. We selected strain AK16^T^ for the whole genome sequencing because there are only two other strains in the genus and only one of them, the pathogenic *G. hollisae* CIP 101886^T^, has been sequenced earlier. The whole genome sequencing of the strain AK16^T^ will help in comparative studies. The strain AK16^T^ also carries some genes which indicate towards pathogenicity. Our main objective in sequencing the genome of AK16^T^ was to compare it with the pathogenic strain to ascertain the differences in the life cycle.

*G. hollisae* CIP 101886^T^ shares a pathogenic gene cluster with *Vibrio* genus. The annotation of the genome of *G. hollisae* CIP 101886^T^ shows 2 out of 5 pathogenic genes reported in some *Vibrio* strains (*Vibrio cholerae* O395). It may be that the other three genes are missing because the genome of *G. hollisae* CIP 101886^T^ is incomplete. Although this suggests the pathogenicity of *G. hollisae* CIP 101886^T^ may be linked with the *Vibrio* genus. Also, we were able to find 11 other genes in the genome of *G. hollisae* CIP 101886^T^ which were a part of *Vibrio* pathogenesis cycle downloaded from the KEGG (Kyoto Encyclopedia of Genes and Genomes) database [10], [11]. Notable point here is that, we found 7 genes of the pathogenicity cycle in the strain AK16^T^ too, which suggests that it may have some interaction with animal host(s) in its life cycle or it may be an opportunistic pathogen.

In this paper, we have discussed the general classification and a set of features for *G. indica* sp. nov. strain AK16^T^ ( = JCM 17852^T^ = MTCC 11632^T^) along with the whole genome sequencing and annotation data. The given data support the circumscription of *Grimontia indica* AK16^T^.

## Materials and Methods

Seawater samples were collected from a southeast coast of Palk Bay (GPS position 9°10′10.10″N 79°25′54.34″E), India. The area falls under Dhanushkodi which is a tourist spot. For collecting coastal waters from this place no permission is required, but there is strict restriction on the sending the samples to abroad. The field studies and the experiment did not involve any endangered or protected species. We had collected the samples and analyzed in India and for this purpose there is no special permission required as we belong to same country. Apart from *G. indica* AK16^T^, two strains of a novel species *Photobacterium marinum*, AK15^T^ and AK18 were also isolated [12]. The water sample was collected and sent in sealed tubes to Institute of Microbial Technology, Chandigarh, India. The strain AK16^T^ was isolated in June 2011 after inoculation on Marine agar 2216 (MA; HIMEDIA, India) in aerobic atmosphere at 30°C.

Strain AK16^T^ was grown aerobically on marine agar plates at 30°C. The DNA of the bacterium was isolated using DNA isolation kit (Zymo Research, California; catalogue number D6005). All reagents and tubes discussed here in DNA isolation protocol were available in the standard kit package, other than unless specified. Bacterial colonies from the plate were picked and mixed with 200 µl of saline phosphate buffer (NaCl 137 mM, KCl 2.7 mM, Na_2_HPO_4_ 10 mM, KH_2_PO_4_ 1.8 mM) in ZR Bashing Bead Lysis Tube. 750 µl of lysis solution was added to the solution and then it was fitted with a 2 ml tube holder assembly. It was then secured in ZR Disruptor Genie (Zymo Research, California; not in the standard kit package) and run for 5 minutes at maximum speed. The assembly was then centrifuged at 10,000×g for 1 minute. Supernatant, around 400 µl, was transferred into ZR Zymo-Spin IV Spin Filter in a collection tube and centrifuged at 7,000×g for one minute. 1,200 µl of ZR Bacterial DNA Binding Buffer was added to the filtrate in the collection tube. 800 µl of the mixture was then transferred to ZR Zymo-Spin IIC column in a collection tube and centrifuged at 10,000×g for one minute. The flow through was discarded and the last step was repeated. The Flow through was discarded again and 200 µl of ZR DNA Pre-Wash Buffer was added to the column after replacing the collection tube and centrifuged at 10,000×g for 1 minute. 500 µl of ZR Bacterial DNA Wash Buffer was added to the column and centrifuged at 10,000×g for 1 minute. The column was then transferred to a clean 1.5 ml microcentrifuge tube and 100 µl of ZR DNA Elution Buffer was added directly to the column matrix and then centrifuged at 10,000×g for 30 seconds to elute DNA.

The 16S rRNA gene sequence was amplified using standard primers. The amplified product was sequenced using Genetic Analyzer ABI 3130XL (Applied Biosystems, California, USA). High quality sequence region was used for BLAST (Basic Local Alignment Search Tool) [13] against 16S rRNA gene sequence based database using EzTaxon-e [14].

The strain AK16^T^, was tested for growth at different temperatures (4, 10, 25, 30, 37, 42, 55°C). Other characteristics were studied as described by Srinivas et al [12]. Growth was checked under anaerobic conditions using Anoxomat Anaerobic System (Mart Microbiology B.V., The Netherlands) and under aerobic conditions. We used VITEK 2 (bioMérieux, Inc., USA) to perform different biochemical assays.

The bacterium was subjected to Matrix-assisted laser-desorption/ionization time-of flight (MALDI-TOF) assay (Bruker Daltonics, Germany) [15]. The test was performed in duplicate using two different colonies from fresh cultures of the bacterium. The data was analyzed using MALDI Biotyper software (version 3, Bruker) to find a match against the main spectra of 3,799 bacteria in the BioTyper database.

Whole genome sequencing of AK16^T^ was performed using two different platforms. Illumina HiSeq 1000 (Illumina Inc., California) platform by C-CAMP (Centre for Cellular and Molecular Platforms, Banglore, India) and in-house using IonTorrent Personal Genome Machine Sequencing (Life Technologies, San Francisco, CA) with 200 bp libraries. Sequencing through Ion-Torrent resulted in 244,444 reads that assembled in 1,599 contigs. Illumina Hiseq platform resulted in 20,166,980 reads that assembled in 130 contigs. Both platform data was used for the hybrid assembly. A total of 20,202,506 high-quality reads with approximately 367x coverage were assembled using CLC Genomics Workbench v. 6.0 (CLC bio, Katrinebjerg, Denmark) (word size 45 and bubble size 65) generating 109 contigs (N50, 119,617 bp) with total length of 5.5 Mbp.

**Figure 3:**
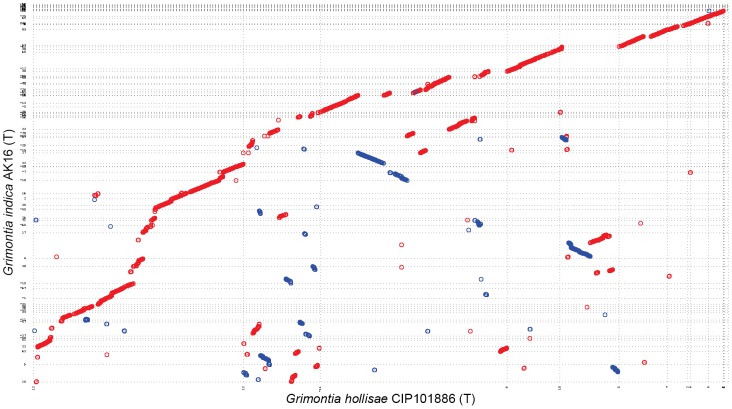
Dot-Plot between *G. hollisae* CIP101886^T^ and strain AK16^T^ created by PROMER from MUMMER package. Red dots represent forward matches while blue dots represents reverse matches.

Annotation of the genome was performed using Rapid Annotation using Subsystem Technology (RAST) pipeline [16]. This pipeline employs GLIMMER3 [17] for gene calling, tRNAscan-SE [18] to identify tRNA genes, and Niels Larsen's “search_for_rnas” (available from the author) to identify rRNA encoding genes. RAST also compares and rectifies positions of proteins using collection of protein families called FIGfams [16]. The protein set was also compared to the National Center for Biotechnology Information (NCBI) non-redundant (nr) database, and COG subset of NCBI Conserved Domain Database (CDD) using NCBI BLAST suit. MUMMER package (http://mummer.sourceforge.net/) was used to create a comparative dot-plot between *G. hollisae* CIP 101886^T^ and AK16^T^ (Figure 3). PROMER was used for creating the plot with default parameters. PROMER translates the nucleotide to amino-acid and then finds the Most Unique Matches (MUMs). The dot-plot hence created is a comparison of translated nucleotide sequence according to the MUMs.

## Results

**Figure 4:**
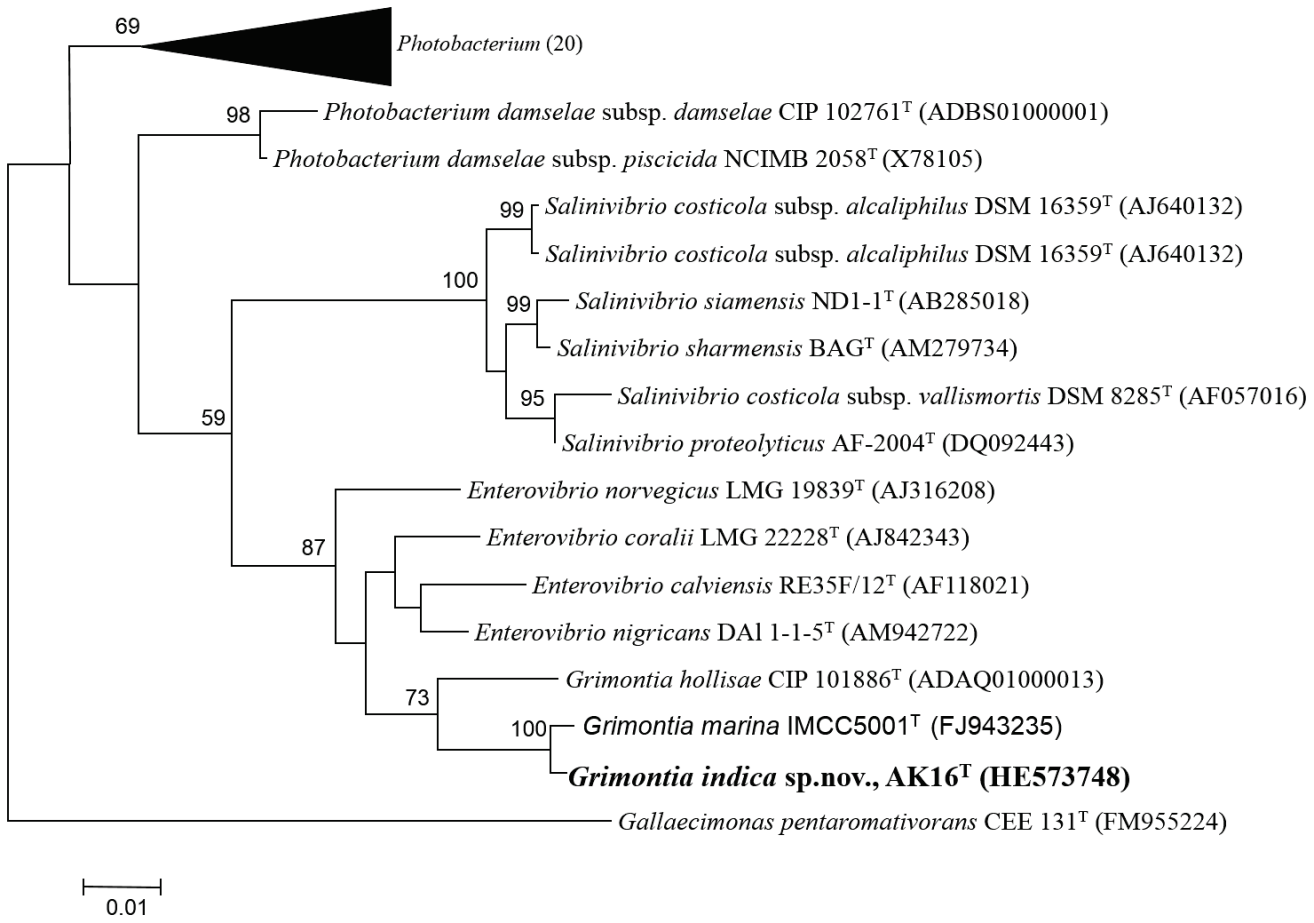
Phylogenetic tree highlighting the position of strain AK16^T^. Phylogenetic tree highlighting the position of strain AK16^T^ relative to other related type strains within the family *Vibrionaceae*. Strains shown are those within the *Vibrionaceae* having corresponding NCBI genome project ids listed within. The tree uses sequences aligned by the Clustal W aligner. The evolutionary history was inferred by using the Maximum Likelihood method based on the Tamura-Nei model [24]. The tree with the highest log likelihood (−5929.7876) is shown. The percentage of trees in which the associated taxa clustered together is shown next to the branches. Initial tree(s) for the heuristic search were obtained automatically as follows. When the number of common sites was <100 or less than one fourth of the total number of sites, the maximum parsimony method was used; otherwise BIONJ method with MCL distance matrix was used. The tree is drawn to scale, with branch lengths measured in the number of substitutions per site. The analysis involved 36 nucleotide sequences. All positions containing gaps and missing data were eliminated. There were a total of 1249 positions in the final dataset. Evolutionary analyses were conducted in MEGA5 [25]. *Gallaecimonas pentaromativorans* CEE 131^T^ (FM955224) was used as an outgroup.

According to the 16S rRNA gene sequence similarity, the nearest phylogenetic neighbors of strain AK16^T^ were “*G. marina* IMCC5001^T^”, with 98.47% similarity, *Enterovibrio coralli* LMG 22228^T^, with 97.03% similarity, *G. hollisae* CIP 101886^T^, with 95.83% similarity, and other strains of genera *Photobacterium*, *Enterovibrio*, *Vibrio* with similarities ranging from 93.43–95.11%. All these similarities were below 98.7% 16S rRNA gene sequence similarity threshold as recommended by Stackebrandt and Ebers [19]. Phylogenetic analyses based on maximum likelihood (ML) tree further indicated that strain AK16^T^ was clustered with *G. hollisae* at a phylogenetic distance of 3.1% and together clustered with species of the genus *Enterovibrio* at a phylogenetic distance of 3.9 to 4.6% and together clustered the species of the genera *Salinivibrio*, and *Photobacterium* (Figure 4). Neighbour joining tree topology was similar to the ML tree (data not shown).

The optimal growth of the bacterium was achieved under aerobic, 25–30°C temperature conditions. While there was slow growth observed under anaerobic conditions. There was no growth at 4 and 55°C, very weak growth at 42°C, and weak growth at 37°C. The colonies were pale yellow, opaque, circular and raised on Marine agar 2216 (HIMEDIA) plates.

Strain AK16^T^ was found positive for catalase and oxidase activity. Many biochemical tests were performed using VITEK 2 (bioMérieux, Inc., USA), in which the strain was found positive for Ala-Phe-Pro-arylamidase, *β*-galactosidase, L-prolinearylamidase, methyl red reaction, indole production, nitrate reduction, and gelatin, Tween 20, and Tween 40 hydrolysis. Strain AK16^T^ is negative for other characteristics (in VITEK 2). Strain AK16^T^ can be differentiated based on some phenotypic characteristics and the different major fatty acid composition (Table 1). All other characteristics of strain AK16^T^ were given in Table S1.

**Table 1:** Differential characteristics of strains *G. hollisae* CIP 101886^T^, *G. marina* IMCC5001^T^, and *G. indica* AK16^T^.

Characteristics	1	2	3
Growth range with NaCl (%)	1–12	1–8	1–6
Growth in anaerobic conditions	+	−	+
Degradation of starch	+	+	−
D-trehalose	+	−	−
D-Mannose	−	+	−
***VITEK 2 GN***			
Indole production	+	−	+
Tween 80 hydrolysis	−	+	+
Aesculin hydrolysis	−	+	−
Urease	+	−	−
Major fatty acids	C_16 : 0_; SF3; SF8	C_16 : 0_; SF3; SF8	C_12 : 0_; C_14 : 0_; C_16 : 0_; SF3; SF8
DNA G+C content (mol%)	51.0	52.6	48.7

Strains: 1, *G. hollisae* CIP 101886^T^; *G. marina* IMCC5001^T^; 3, *G. indica* AK16^T^.

Data 1 and 2 are from previously published data [26], data 3 was experimentally ascertained. All three strains did not degrade casein. Also, all of these strains did not produce H_2_S gas.

# SF, summed features; Summed features represent groups of two or three fatty acids that cannot be separated by GLC with the MIDI system. Summed feature 3 contains C_16: 1*ω7c*_/iso-C_15 : 0_ 2OH; summed feature 8 containsC_18:1ω6c_ and/or C_18:1ω7c_.

**Figure 5:**
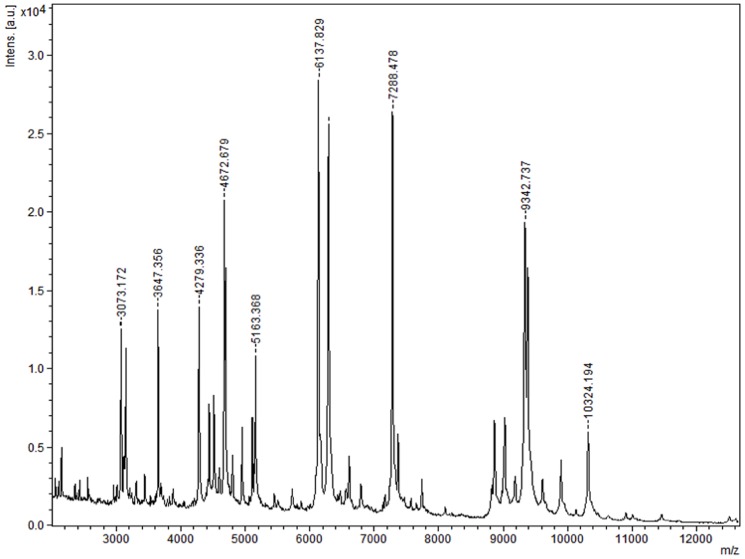
MALDI-TOF reference mass spectrum from strain AK16^T^. MALDI-TOF reference spectrum with peak weights is illustrated. No significant similarities were reported.

The MALDI-TOF analysis reported no significant matches for the strain AK16^T^, suggesting it to be a novel strain as far as Bruker database is concerned. The average identification score value was 1.317, which was below the threshold of 1.9 required to announce identity. The reference spectrum is illustrated in Figure 5 with labeled peak weights. Although, the absence of other *Grimontia* spectra does not yet make MALDI-TOF MS a discriminative identification criterion for strain AK16^T^.

### Genome properties of strain AK16^T^

The draft genome consists of one incomplete circular chromosome of 5,555,590 bp (48.7% G+C content) in 109 contigs. Total 4,956 genes were predicted, 3,658 of which are protein-coding genes. 2,378 of protein coding genes were assigned to a putative function with the remaining annotated as hypothetical proteins. The draft genome of *G. hollisae* CIP101886^T^ consists of an incomplete circular chromosome of 4,002,786 bp (49.47% G+C content) in 13 contigs. A total of 3,777 genes were predicted out of which 3,651 genes were protein coding genes while 126 were RNA genes. Out of the total 3,651 protein coding genes, 2,779 were assigned some putative function while others were annotated as hypothetical proteins. A detailed comparison of the genomes is given in table 2 and 3.

**Table 2:** Nucleotide content and gene count levels of the genome.

Attribute	Genome (total)			
	*Grimontia indica* AK16^T^		*Grimontia hollisae* CIP 101886^T^	
	Value	% of total^a^	Value	% of total^a^
Size (bp)	5,555,590	100.00	4,002,786	100.00
G+C content (bp)	2,705,753	48.70	1980305	49.47
Coding region (bp)	4,742,892	85.37		
Total genes	4,956	100.00	3,777	100.00
RNA genes	87	1.76	126	3.33
Protein-coding genes	3,658	73.80	3651	96.66
Genes assigned to COGs	4,603	92.90	3439	91.05
Genes with signal peptides	1,301	25.78	860	22.76
Genes with transmembrane helices	1,358	26.90		
CRISPR repeats	1	0.02	1	0.03

a) The total is based on either the size of the genome in base pairs or the total number of protein coding genes in the annotated genome.

**Table 3:** Number of genes associated with the 25 general COG functional categories.

Code	*Grimontia hollisae* CIP 101886^T^		*Grimontia indica* AK16^T^		Description
	Value	% of total^a^	Value	% of total^a^	
J	201	4.37	174	5.06	Translation
A	2	0.04	2	0.06	RNA processing and modification
K	372	8.08	231	6.72	Transcription
L	154	3.35	170	4.94	Replication, recombination and repair
B	2	0.04	1	0.03	Chromatin structure and dynamics
D	42	0.91	44	1.28	Cell cycle control, mitosis and meiosis
Y	0	0	0	0.00	Nuclear structure
V	71	1.54	43	1.25	Defense mechanisms
T	393	8.54	276	8.03	Signal transduction mechanisms
M	268	5.82	216	6.28	Cell wall/membrane biogenesis
N	127	2.76	137	3.98	Cell motility
Z	0	0	0	0.00	Cytoskeleton
W	0	0	0	0.00	Extracellular structures
U	101	2.20	119	3.46	Intracellular trafficking and secretion
O	170	3.70	135	3.93	Posttranslational modification, protein turnover, chaperones
C	247	3.37	199	5.79	Energy production and conversion
G	324	7.04	212	6.16	Carbohydrate transport and metabolism
E	424	9.21	289	8.40	Amino acid transport and metabolism
F	99	2.15	82	2.38	Nucleotide transport and metabolism
H	178	3.87	153	4.45	Coenzyme transport and metabolism
I	140	3.04	98	2.85	Lipid transport and metabolism
P	226	4.90	178	5.18	Inorganic ion transport and metabolism
Q	115	2.50	59	1.72	Secondary metabolites biosynthesis, transport and catabolism
R	534	11.60	340	9.89	General function prediction only
S	413	8.97	281	8.17	Function unknown
-	444	9.65	338	9.83	Not in COGs

a) The total is based on the total number of protein coding genes in the annotated genome.

## Discussion

**Figure 6:**
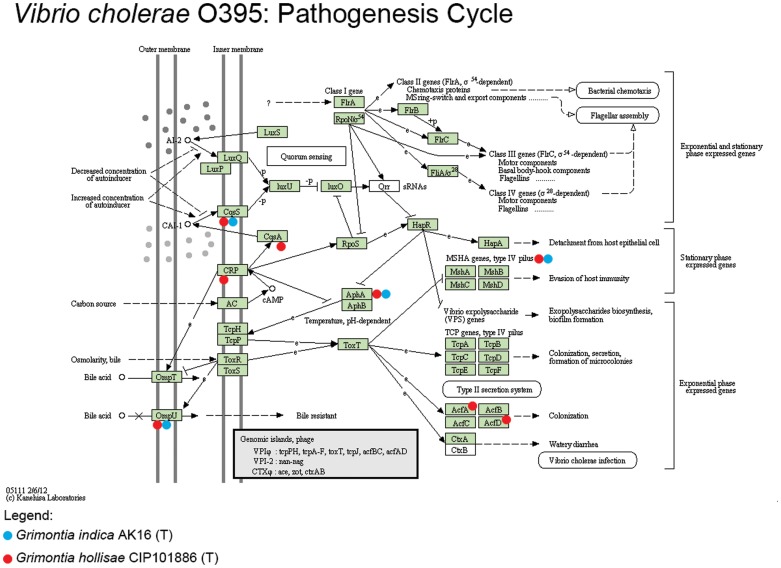
*Vibrio cholerae* O395 pathogenesis cycle downloaded from KEGG database. Genes in the green rectangle are found in *Vibrio cholera* O395 and are a part of the pathogenesis cycle. Red and Blue coloured dots denote the presence of that gene in strain AK16^T^ and *G. hollisae* CIP101886^T^ respectively.

Apart from strain AK16^T^, only one other bacterium, *G. hollisae* CIP101886^T^, under the genus *Grimontia* has whole genome sequence data available. The smaller, 4,002,786 bp long draft genome of *G. hollisae* CIP101886^T^ has 13 contigs, with slightly higher, 49.47%, G+C content when compared to strain AK16^T^, a detailed comparative data is illustrated in table 2. RAST reports *G. hollisae* CIP101886^T^ to be the nearest neighbor of strain AK16^T^ with similarity score value of 525, followed by *Vibrio* sp. Ex25, *Vibrio parahaemolyticus* RIMD 2210633, and *Vibrio angustum* S14 with similarity score values 397, 350, and 280 respectively. The genome of *G. hollisae* CIP101886^T^ was again annotated using the same pipeline as used for strain AK16^T^ for a fair comparison. The new annotation of *G. hollisae* CIP101886^T^ has 3,777 genes out of which 3,439 (91.05%) were assigned to COG categories, which is little less than strain AK16^T^ (92.90%), a detailed comparison can be found in table 3. There were two adhesion genes, AcfA, and AcgD, present in *G. hollisae* CIP101886^T^ that play role in chemotaxis by assisting in intestinal colonization which aids in pathogenesis of the bacterium. These genes were absent in the genome of strain AK16^T^, which hints a non-pathogenic life cycle in seawater. These two genes, AcfD and AcfA, are part of a total 5 gene cluster present in some *Vibrio cholera* strains like *Vibrio cholera* O395. The missing genes may be present in the genome of *G. hollisae* CIP101886^T^ but due to the incomplete draft sequence of the genome, the genes were missed. Further, we downloaded the KEGG pathogenesis cycle for *Vibrio cholera* O395 (Figure 6) and searched for the pathogenesis genes of the cycle in the genome of *G. hollisae* CIP101886^T^ and AK16^T^. Total 11 genes were found matching with *G. hollisae* CIP101886^T^ while 7 genes were found in AK16^T^. In case of *G. hollisae* CIP101886^T^ there were some unique matches like the CRP gene which is involved in production of HapR gene. This HapR gene is the major regulator of quorum sensing and virulence [20]. There were CqsA and CqsS (CqsS is also present in AK16^T^) genes which are involved in quorum sensing. The AphA and LuxR genes found in both the strains work under synchronization to ensure maximum LuxR/HapR production at low cell density [21]. OmpU gene found in both the strains is the key determinant of pathogenic interactions with the host. OmpU acts as adhesion/invasion required for *β*-integrin recognition and host cell invasion [22]. Also, there are multiple Mannose-Sensitive Haemagglutinin (MHSA) biogenesis proteins, namely, MshI, MshK, MshE, MshC, MshO, MshQ, MshH, and MshD present in the genome of both the strains. MSHA is a surface pilus which helps in adhesion to host surface [23].

Although, *G. hollisae* CIP101886^T^ carried more pathogenic genes than AK16^T^, it may be that AK16^T^ missing those genes in the whole genome sequencing as it carries much more contigs (109) than the *G. hollisae* CIP101886^T^ genome (13). This indicates that the strain AK16^T^ may be a pathogen to some marine animal(s).

Dot-plot between *G. hollisae* CIP101886^T^ and strain AK16^T^ created using PROMER from the MUMMER package (Figure 3) shows a discontinuous diagonal line with slight deviations. This tells us that *G. hollisae* CIP101886^T^ and strain AK16^T^ may share a common ancestor.

### Conclusion

The phenotypic, phylogenetic and genomic analyses of strain AK16^T^ suggest it to be a novel species under the genus *Grimontia* and hence we formally propose the creation of *G. indica* sp. nov.that contains the strain AK16^T^. This bacterium was isolated from a seawater sample collected from Palk Bay, India.

The strain AK16^T^ carries some genes that are related to the pathogenic cycle of *Vibrio cholerae* and other *Vibrio* species. This suggests us that the bacterium may be having pathogenic interactions with some animal host as part of its life cycle. It can also be that the bacterium may be an opportunistic pathogen. This gives rise to a hypothesis that the other strain, ‘*G. marina* IMCC5001^T^’ also isolated from seawater sample may carry some pathogenic traits and that all strains of *Grimontia* genus may be pathogenic. Further studies may be conducted to work on the hypothesis.

### Description of *Grimontia indica* sp. nov

*Grimontia indica* (in'di.ca. L. fem. adj. indica, of India, Indian).

The colonies of the bacterium AK16^T^ were raised, opaque, pale yellow, 1 mm in diameter on Marine Agar plates. Cells were rod shaped. The bacterium grows optimally under aerobic conditions. Growth was also observed under anaerobic conditions. Growth of the bacterium occurs between temperature range of 10–42°C, with optimal temperature between 25–30°C, pH range of 6–12, with optimum pH 7, and required salt concentration in the range of 2–6% NaCl. The cells are Gram-negative, non-endospore forming, and motile. The bacterium is positive for catalase, oxidase, Ala-Phe-Pro-arylamidase, *β*-galactosidase, L-prolinearylamidase, methyl red reaction, indole production, nitrate reduction, and gelatin, Tween 20 and Tween 40 hydrolysis. The G+C content of the genome was 48.7%. The 16S rRNA gene sequence and whole genome sequence of the bacterium are submitted in GenBank with the accession numbers HE573748 and ANFM00000000 respectively. The type strain AK16^T^ ( = JCM 17852^T^ = MTCC 11632^T^) was isolated from a seawater sample collected from Palk Bay, India.

## Supporting Information

Table S1 Different phenotypic characteristics of strain AK16T.
(PDF)
