# Supplementary material for: Grimontia indica AK16T, sp. nov., Isolated from a Seawater Sample Reports the Presence of Pathogenic Genes Similar to Vibrio Genus
Source: PLoS One. 2014 Jan 21;9(1):e85590. doi: 10.1371/journal.pone.0085590 (PMC3897461; doi:10.1371/journal.pone.0085590)
Supplement: Table S1 — Different phenotypic characteristics of strain AK16T. (PDF) [file pone.0085590.s001.pdf]

Table S1. Different phenotypic characteristics of strain AK16^T

Characteristics	*Grimontia indica* AK16^T
Cell Morphology	Rods
Motility	Motile
Flagellum	Single polar
Catalase	+
Oxidase	+
VITEK 2 GN	
Ala-Phe-Pro-arylamidase	+
Adonitol	-
L-pyrrolydonyl-arylamidase	-
L-arabitol	-
D-cellobiose	-
β-galactosidase	+
H_2S production	-
β-*N*-acetylglucosaminidase	-
Glutamyl arylamidase	-
D-glucose	-
γ-glutamyl-transferase	-
Fermentation glucose	-
β-glucosidase	-
D-maltose	-
D-mannitol	-
β-xylosidase	-
β-alanine arylamidase	-
L-proline arylamidase	+
Lipase	-
Palatinose	-
Tyrosine arylamidase	-
D-sorbitol	-
Sucrose	-
D-tagatose	-
Sodium citrate	-
Malonate	-
5-keto-D-gluconate	-
L-lactate alkalinisation	-
α-glucosidase	-
Succinate alkalinisation	-
β-*N*-acetylgalactosaminidase	-
α-galactosidase	-
Phosphatase	-
Glycine arylamidase	-
Ornithine decarboxylase	-
Lysine decarboxylase	-
L-histidine assimilation	-
Courmarate	-
β-glucoronidase	-

Characteristics	*Grimontia indica* AK16^T
0/129 resistance	-
glu-gly-arg-arylamidase	-
L-malate assimilation	-
ELLMAN	-
L-lactate assimilation	-
Physiological tests	
Temperature	
4ºC	-
10ºC	W
25ºC	+
30ºC	+
37ºC	W
42ºC	VW
55ºC	-
pH	
5	-
6	+
7	+
8	+
9	+
10	+
11	+
12	+
NaCl	
2%	+
4%	+
6%	+
8%	-
10%	-
12%	-
Substrate hydrolysis	
Casein	-
DNA	-
Gelatin	+
Tween 20	+
Tween 40	+
Tween 60	+
Biochemical	
Arganine dihydrolase	-
Methyl red	+
Voges Proskeur	-
Nitrate reduction	+
Citrate utilization	-
Growth on MacConkey agar	-

+, positive; -, negative; W, weak growth; VW, very weak growth.
